# Does Superior Semicircular Canal Dehiscence Surgery Resolve or Exacerbate Positional Vertigo?

**DOI:** 10.3390/audiolres15060165

**Published:** 2025-11-28

**Authors:** Gerard Joseph Gianoli

**Affiliations:** Ear and Balance Institute, 1401 Ochsner Blvd, Covington, LA 70433, USA; ggianoli@gmail.com; Tel.: +1-985-809-1111

**Keywords:** Benign Paroxysmal Positional Vertigo, Superior Semicircular Canal Dehiscence, canalith repositioning, Epley maneuver, surgery

## Abstract

Does Superior Semicircular Canal Dehiscence Surgery Resolve or Exacerbate BPPV? **Background/Objectives:** BPPV is commonly found to be associated with other inner ear disorders. It has been found to occur with Superior Semicircular Canal Dehiscence (SSCD) as well as postoperatively following SSCD surgical repair. This paper will analyze the preoperative and postoperative incidence of positional vertigo in patients undergoing SSCD surgery. **Methods:** This is a retrospective chart review of 50 consecutive patients with SSCD undergoing surgical repair. They were evaluated preoperatively, at 1 week postoperative, at 6 weeks postoperative, and at 12 weeks postoperative for evidence of BPPV. Information collected included demographics, the semicircular canal involved, type of BPPV, and whether the patient required canalith repositioning. **Results:** Preoperatively, 33 (66%) patients reported symptoms of positionally induced vertigo with confirmation during VNG testing. No patient was treated for BPPV prior to surgery. At one week postoperative, 17 (35%) patients continued to have symptoms of positionally induced vertigo; at 6 weeks postoperative, 9 (18%), and at 12 weeks postoperative, 5 (10%) patients had positional vertigo requiring canalith repositioning (*p* < 0.05). Three patients (6%) had no evidence of BPPV preoperatively but had positional vertigo at the one-week postoperative evaluation. At the 6-week post-op visit, only one of the patients had new-onset postoperative positional vertigo. At the 3-month visit, no patient had new-onset postoperative positional vertigo. **Conclusions:** BPPV and positional vertigo symptoms were found commonly prior to SSCD surgery and in the week after SSCD surgery. However, BPPV resolved by 6 weeks after SSCD surgery without additional intervention for most of these patients, while the others underwent canalith repositioning. A small percentage developed BPPV after surgery who had none preoperatively and in the contralateral ear.

## 1. Introduction

Since John Epley introduced the Epley canalith repositioning maneuver as a means for treatment of Benign Paroxysmal Positional Vertigo (BPPV), BPPV has become the most identified cause of vertigo [1]. BPPV has been suggested to develop due to the dislocation of utricular otoconia. The Epley maneuver is a physical maneuver to reposition the otoconia from the posterior semicircular canal to the vestibule. The Epley maneuver has been, arguably, the single biggest advance in vestibular medicine in our lifetime. Multiple other repositioning maneuvers have been developed for repositioning the otoliths from other semicircular canals and for the variants of canalithiasis and cupulolithiasis [2]. However, as successful as these maneuvers may be, they do not answer the question of why the otoconia have become dislodged initially or recurrently.

Superior Semicircular Canal Dehiscence (SSCD) is defined as the absence of bone over the superior semicircular canal, where it interfaces with the middle fossa dura (or the superior petrosal sinus) [3,4]. SSCD can cause a variety of symptoms and has been nicknamed a “Great Otologic Mimicker” due to its ability to mimic other ear pathologies [5]. However, the most characteristic symptoms are Tullio phenomenon, strain-induced vertigo/dizziness, pulsatile tinnitus, conductive hyperacusis, and autophony. Surgical repair of SSCD has been demonstrated to reliably relieve the symptoms of SSCD [6].

BPPV has been associated with trauma and other inner ear pathologies. Specifically, BPPV has been associated with SSCD and after SSCD surgery [7]. Given the abnormal utricular stimulation (i.e., oVEMP testing), this would seem to be expected. It has been proposed that SSCD can be an integral cause of the dislodgement of otoconia due to the pressure effects on the utricle [8]. SSCD has also been observed to cause positional nystagmus, which is presumably not due to loose otoconia but due to the differential pressure effects on the dehiscent site during Dix–Hallpike testing [9,10]. This consideration has led to the authors recommending SSCD as a potential etiology for recurrent BPPV. This paper will review the incidence of BPPV before and after SSCD surgery.

## 2. Methods

This is a retrospective chart review of 50 consecutive patients who underwent SSCD surgery. Inclusion criteria included patients with a diagnosis of SSCD and who had undergone surgical repair for SSCD. The diagnosis of SSCD was made according to the Barany Society criteria [11]. All patients underwent history (including questioning regarding positionally induced vertigo/dizziness) and extensive vestibular testing, including Dix–Hallpike testing. Demographics and outcomes from history and Dix–Hallpike testing were recorded. The Dix–Hallpike test was performed with infrared video goggles and required concomitant symptoms of vertigo/dizziness and appropriate nystagmus. Some underwent additional analysis with the Epley Omniax. The diagnosis of BPPV was made according to the Barany Society criteria [12].

At the one-week postoperative visit, the patients were assessed for symptoms of positionally induced vertigo/dizziness, but discreet testing was not performed at that time. At the 6-week postoperative visit, the patients were questioned regarding the occurrence of positionally induced vertigo/dizziness since surgery. Any patient with persistent positionally induced vertigo/dizziness was assessed with the Dix–Hallpike exam and/or evaluation in the Epley Omniax. Canalith repositioning was performed as deemed appropriate. At the 3-month follow-up, patients were again assessed for positionally induced symptoms of vertigo/dizziness. Any patients with positionally induced symptoms were evaluated with the Dix–Hallpike or Epley Omniax.

The surgical procedure employed for SSCD was a capping procedure via a combined middle fossa/transmastoid approach using calvarial bone and silastic sheeting for coverage over the dehiscent site. Concomitantly, round and oval window reinforcement was also performed with temporalis fascia and fibrin glue. Further details on the specifics of surgical intervention are reported elsewhere [13].

Comparison of positionally induced vertigo/dizziness preoperatively and then at 1 week, 6 weeks, and 12 weeks postoperatively was performed. The outcomes were statistically analyzed using the McNemar chi-square analysis. The procedures followed were in accordance with the ethical standards of the responsible committee on human experimentation and with the Helsinki Declaration. The Salus Institutional Review Board approved this study.

## 3. Results

All 50 patients underwent the above-described surgery; one patient underwent a concomitant type I tympanoplasty and another patient underwent a concomitant middle fossa encephalocele repair.

Among the 50 patients, 35 were female and 15 were male. The average age was 46 years, with a range of 15–74 years. Among the patients, 48 (96%) had primary complaints of vertigo or disequilibrium. Only two patients (4%) underwent surgery for the primary complaint of autophony or conductive hyperacusis, although they also noted vestibular symptoms. Data on positional symptoms was available for all patients preoperatively and 1 week and 6 weeks postoperatively. There was data for 49 of the 50 patients at the 12 week follow-up.

Preoperatively, 33 of the 50 patients (66%) reported symptoms of positionally induced vertigo or dizziness. Among these patients, testing revealed 24 with posterior canalithiasis, 2 with a horizontal canal variant, and 7 with an anterior canal variant. All were canalithiasis BPPV, and no cupulolithiasis cases were identified. No patient was treated for BPPV preoperatively.

At the 1-week postoperative visit, 17 patients (34%) endorsed positionally induced vertigo/dizziness. This included three patients who had no symptoms of positionally induced vertigo preoperatively. No intervention was deployed (i.e., canalith repositioning) at this time.

At the 6-week postoperative visit, there were only nine patients (18%) with symptoms of positionally induced vertigo. All had posterior canalithiasis and were subsequently treated with canalith repositioning in the Epley Omniax multiaxial device. Two of the three patients who had new-onset positional vertigo at the 1-week post-op visit had resolution without intervention by the 6-week post-op visit. Among those with BPPV at the 6-week visit, there were two cases of BPPV contralateral to the operative ear and one case of bilateral posterior canal BPPV.

At the 3-month visit, only five patients had positional vertigo. All of these patients had been identified as having BPPV preoperatively. In other words, there were no new-onset postoperative cases of BPPV. Two of these patients developed positionally induced vertigo after the 6-week visit (i.e., no positional symptoms at the time of the 6-week visit). All had BPPV ipsilateral to the surgical ear. Four had posterior canal involvement and one had anterior canal involvement. Only one patient did not have follow-up data at the 12 week follow-up period.

Statistical analysis revealed a statistically significant (*p* < 0.05) difference in the number of patients with BPPV for each successive postoperative visit compared to preoperatively, as well as the subsequent postoperative visits (see Table 1).

## 4. Discussion

To date, there has been little investigation in the medical literature regarding vertigo or dizziness provoked by positional changes in SSCD patients. SSCD has been defined by its most characteristic symptoms, which include autophony, conductive hyperacusis, strain-induced vertigo, and Tullio phenomenon. In 2017, Naert and colleagues [14] reported on an aggregation of symptoms in SSCD reported in 431 patients in 66 articles. The five most common symptoms reported were spontaneous dizziness (51%), sound-induced vertigo (42.7%), autophony (42.5%), pressure-induced vertigo (37.4%), and hearing loss (39.9%). Positionally induced vertigo as a symptom was much less frequently noted—only identified in 1.6% of patients.

While not traditionally seen as a common symptom of SSCD, BPPV was found to occur commonly after SSCD surgery by Barber et al. [7]. In their retrospective study, they compared 84 surgical SSCD patients with 94 non-surgical SSCD patients. The postoperative occurrence of BPPV was 23.8% compared to 6.2% in the non-operative patients. They noted the majority of postoperative BPPV occurred during the first 3 months after surgery.

Conversely, Xie et al. [15] reported on postoperative complications of SSCD surgery and, although BPPV was not the main focus of their retrospective study, they identified BPPV as a postoperative complication in 4.5% of 242 SSCD surgeries.

The present study specifically evaluates positionally induced symptoms and positional testing in 50 patients undergoing SSCD surgery and at three postoperative visits. The findings are different from prior reports in that the symptoms of positionally induced vertigo were quite high (66%) preoperatively and corroborated by positional testing, identifying BPPV. Whereas the two prior reports identify BPPV as a complication of surgery in 4.5% and 23.8% of patients after SSCD surgery, we found a lower incidence of d BPPV postoperatively compared to preoperatively. That said, by the 12 week postoperative visit, the incidence of BPPV was only 10% which compares favorably with the two prior reports of 4.5% and 23.8% incidence of postoperative BPPV.

The most striking finding of this survey is that, as opposed to SSCD surgery causing BPPV as a complication, it appears to result in a lower incidence of BPPV. This raises the possibility that SSCD may have been somehow involved in the propagation of BPPV for these patients. In fact, although not specifically addressed in this study or systematically analyzed, many of these patients presented to the author because of longstanding recurrent BPPV rather than typical symptoms of SSCD. This is likely the reason that the incidence of BPPV preoperatively in this study is much higher than in prior studies. This study also raises the question as to whether recurrent BPPV in a patient with SSCD can be controlled with surgery for SSCD. In 2019, Pirodda and Brandolini [8] proposed that the pressure effects of SSCD on the inner ear, and presumably the utricle, could result in the facilitation of otoconial release. This theory could explain the high incidence of BPPV seen in the present series of patients. While further research is warranted to investigate this in a more prospective analysis, this retrospective study seems to support such a notion. Further research can address whether the reason for the resolution of BPPV postoperatively is the elimination of the pressure effects seen in SSCD or some other factor. Another interesting finding of this study is the high incidence of anterior canal BPPV noted preoperatively (14%), with the resolution of all but one case postoperatively without canalith repositioning. Anterior canal BPPV has been identified as a postoperative complication of SSCD surgery, but not preoperatively [16]. The high incidence of anterior canal BPPV might be explained by the endolymphatic pressure effects on the anterior canal cupula. In 2019, Young et al. [9] presented a case of positionally induced vertigo in an SSCD patient that mimicked anterior canal BPPV, which failed to resolve with repositioning maneuvers. They concluded the positional vertigo was a result of pressure effects from SSCD due to positional changes. Similarly, in 2014, Kundaragi et al. [10] reported a case of a patient with dehiscences of both superior and posterior canals presenting with symptoms of positional vertigo. They emphasized the importance of including SSCD in the differential diagnosis of a patient with atypical or persistent positional vertigo.

Another potential explanation for the high incidence of anterior canal BPPV in this study is the possibility that some participants had apogeotropic posterior canal BPPV. This was not effectively reported in the medical records and thus cannot be commented upon. This should be addressed in future studies. One technique to help distinguish anterior canal BPPV from posterior canal apogeotropic BPPV would be the use of vHIT, as espoused by Castelucci et al. [17].

The weaknesses of this study are those typical of a retrospective study and its short follow-up time. Longer follow-up durations are needed to see if this effect on positional vertigo is sustained beyond 12 weeks, or whether this represents a short-term fluctuation of BPPV. While the spontaneous resolution of BPPV after surgery is possible, the declining incidence of BPPV at the 1-week, 6-week, and 12-week postoperative visits suggests a causal relationship between surgical repair and reduced BPPV incidence. While the pre-op visit included objective testing, the 1-week visit did not include Dix–Hallpike testing but only patient-reported symptoms of BPPV, which could have altered the outcomes of this study. The 6-week and 12-week visits only included testing if a patient was symptomatic of positional vertigo/dizziness, unlike the preoperative visit, which included objective testing of all patients. This could also alter the outcomes of the study. However, it should be noted that all patients who preoperatively complained of positional vertigo were objectively identified as having positional nystagmus upon testing. Furthermore, the symptomatic resolution/persistence of positional vertigo was assessed in all patients. A prospective study with objective testing in all patients would be more accurate and comprehensive.

## 5. Conclusions

Preoperative symptoms of positional vertigo/dizziness were common among this group of patients undergoing surgery for SSCD (66%). The incidence of patients having positionally induced symptoms, while still high 1 week after surgery, was less than at the preoperative stage. By the 6-week postoperative visit, most cases of BPPV had resolved without canalith repositioning or other interventions for BPPV. By the 3-month postoperative visit, only 10% had any residual positional vertigo. While spontaneous resolution of BPPV is possible, the surgical repair of SSCD may have a positive effect on BPPV resolution.

## Figures and Tables

**Table 1:** Incidence of Positional Vertigo Preoperatively and Postoperatively.

	Preop	1-wk *	6-wk	12-wk
Preoperative Positionally Induced Vertigo/Dizziness	33 (66%)	17 (34%)	9 (18%) **	5 (10%) ***
Posterior Canalithiasis	24	NA	9	4
Horizontal Canalithiasis	2	NA	0	0
Anterior Canalithiasis	7	NA	0	1

Number of patients (percent of 50 total) of patients who endorsed symptoms of positionally induced vertigo/dizziness preoperatively and 1 week, 6 weeks, and 12 weeks postoperatively. (*p* < 0.05) * Dix–Hallpike testing was not performed during the 1-week postoperative visit. ** One patient had BPPV contralateral to the surgical ear and one patient had bilateral BPPV at the 6-week visit. *** There were two patients who had new-onset BPPV at the 12-week visit. NA—not assessed at that time.

## Data Availability

The original data presented in the study are included in the article, further inquiries can be directed to the corresponding author.
